# Exploring the co-involvement of disabled adolescents in participatory action research; protocol for a critical interpretative synthesis

**DOI:** 10.12688/hrbopenres.13343.1

**Published:** 2021-07-22

**Authors:** Fiona McDonald, Katie Robinson, Aoife L. Gallagher, Judith Pettigrew

**Affiliations:** 1 School of Allied Health, Health Research Institute, University of Limerick, Limerick, V94 T9PX, Ireland

**Keywords:** Critical Interpretative Synthesis, Participatory Action Research, Adolescents, Disabled, Neurodevelopment

## Abstract

**Background:** Participatory action research (PAR) provides an opportunity for academic researchers and adolescents to co-conduct research within an area of shared interest. Reciprocal learning occurs as co-researchers acquire research skills and knowledge, and academic researchers gain understanding of the issue being examined, from the perspective of those with lived experience. All members of the research team have a shared responsibility for the research and decision-making processes. PAR has predominantly involved adults as co-researchers. However, in recent years more effort has been made to co-conduct research with adolescents. The aim of this review is to interrogate the practices of academic researchers employing a PAR approach when working along-side disabled adolescents.

**Methods/design:** A critical interpretive synthesis (CIS) will be conducted, allowing for a diverse range of evidence to be drawn from. A systematic search of nine databases, from 1990 onwards, will be conducted first. Reference checking will occur to elicit further relevant data. Following screening, further purposive sampling will be completed to facilitate the development of concepts and theory in line with the on-going analysis and synthesis of findings. Data analysis will involve interpretation of included papers in relation to the principles of PAR and a ‘best-practice’ framework will be developed. During analysis particular emphasis will be given to the identification of potential social barriers to the participation of disabled adolescents in PAR.

**Discussion:** PAR is widely employed but little is known about its use when working with disabled adolescents. This current CIS will critically question the current practices of academic researchers employing PAR when working along-side disabled adolescents and future research through the best practice framework we will develop.

## Introduction

It has long been established, in policy and legislation, that children and young people should be given influence in decisions about their lives ( Christensen, 2004; UN, 1989). This is particularly the case for disabled adolescents who are often marginalised ( Kembhavi & Wirz, 2009). Since the UN Convention of Rights of the Child ( UN, 1989), it has become widely accepted that young people should be supported to share their perspectives and to have these attended to by others ( Kennan *et al.,* 2018). A partnership approach is also an expectation in research ( Larsson *et al.,* 2018; Lundy *et al.,* 2011). Participatory action research (PAR) provides adolescents with a collaborative forum to address issues that impact them and their peers, to share opinions and expertise within the research process and to partner in the identification of priority areas for research ( Kirby, 2004).

Within PAR, it is expected that co-researchers are actively involved at all stages of the research process ( Greenwood *et al.*, 1993), working alongside the academic researcher, as an expert in their own right ( Anselma *et al.*, 2019; Baum, 2016). Reciprocal learning occurs as co-researchers acquire additional skills and knowledge, such as research skills, and the academic researcher benefits from an increased understanding of the condition or issue being examined from the co-researcher perspective ( Blair & Minkler, 2009).

Three key principles of PAR have been identified by Rodriguez & Brown (2009), evolving from their work in co-conducting research with youth. These include the need to establish the area of inquiry in line with the authentic life experience of the co-researchers, to engage in collaborative practices which minimise barriers to participation, and to attend to the transformative nature of PAR, ensuring action points to tackle the social injustices experienced. Additionally, five integral processes have been described within PAR: the requirement for co-researchers to be trained and practised in research; provision of opportunities for discussion and shared problem-solving; an iterative approach to analysis and action; the development of ancillary networks over time; and power-sharing within the research team ( Ozer & Douglas, 2015).

Although shared decision-making and responsibility for the research are deemed essential components of PAR ( McTaggart, 1991), in practice, power imbalances often occur ( Jacquez *et al.*, 2013; Willumsen *et al.*, 2014). Particular challenges have been noted when adult researchers work alongside younger researchers, which has been attributed to the ethical responsibility of the adult to care for the well-being of the younger person. This highlights the need for the adult researcher to proactively adopt a reflexive approach to facilitation ( Call-Cummings *et al.*, 2020).

This protocol uses the language of ‘disabled adolescents’ versus the person-first approach often used in health research (adolescents with a disability). This decision reflects our view that disability cannot be attributed purely to individual characteristics and recognises the societal influences in the experience of disability ( Degener, 2016; Shakespeare, 2006). The term ‘disability’ is a contested one, with multiple definitions; indeed, attainment of a single agreed definition is unlikely due to the complex nature and needs of society ( Iezzoni & Freedman, 2008). 

Perspectives on disability have evolved over time from an early religious model ( Eerola, 2012) , to a medicalised model of disability ( Barnes, 1997) to social models of disability ( Shakespeare & Watson, 1997). The medical model situates ‘the problem’ of disability with the person, constructing the impairment as an abnormality ( Berghs *et al.*, 2016), which requires the intervention of health professionals ( Snoddon & Underwood, 2014). The medical model of disability was challenged by disability activists in the 1970’s ( Tregaskis, 2002), creating the conditions for the social model of disability to emerge. The social model of disability situates ‘the problem’ firmly in social oppression through environmental, cultural and attitudinal barriers, which preclude disabled person’s equal participation in society ( Abberley, 1987; Dirth & Branscombe, 2017; Oliver, 2013).

Many studies have determined that disabled persons are impacted by both the underlying impairment and social barriers to participation ( Broersma *et al.*, 2018; Schulz *et al.*, 2012; Shakespeare, 2006; Shields *et al.*, 2012). Acknowledging this, the social relational model of disability, developed by Thomas (2004), will guide this review. This model accepts that impairments and chronic health conditions can negatively impact function but “ *disability only comes into play when the restrictions of activity experienced by people with impairment are socially imposed”* ( Thomas, 2004).

Historically PAR has been used to address such marginalisation by providing a safe space for collaboration that engenders meaningful change at a policy or practice level ( Brydon-Miller *et al.*, 2020). This is reflected in the guiding definition of PAR, to be operationalised within this CIS, which describes PAR as “an *empirical methodological approach in which people directly affected by a problem under investigation engage as co-researchers in the research process, which includes action, or intervention, into the problem*” ( Rodríguez & Brown, 2009). This review will interrogate how the principles of PAR are employed in practice, determining the extent to which they are operationally applied. We will draw on the ‘best practice’ principles, described by Israel *et al.* (2010), in relation to community-based participatory research (CBPR), which have since been used in multiple studies ( Blair & Minkler, 2009; Cargo & Mercer, 2008; Christopher *et al.*, 2008; Nicolaidis *et al.*, 2019; Wallerstein & Duran, 2010). The core philosophies of CBPR are shared by other research approaches situated within the umbrella term of ‘participatory research’, including PAR ( Cargo & Mercer, 2008). These principles pertain to: (a) collaboration; (b) shared learning; (c) empowerment, including the provision of accessible and needs-driven training and facilitation processes; (d) reflexivity by the research team, with feedback loops and resultant adaptation of processes; (e) meaningful change through action and capacity building; and lastly (f) employment of a partnership approach which respects and optimises the knowledge and expertise of different stakeholders ( Israel *et al.,* 2010).

From the existing literature, it is evident that challenges occur in employing PAR principles; two reviews have specifically identified shortcomings in the use of PAR with older adults. Blair and Minkler’s review in (2009) found only a small number of exemplary studies (n=10) that involved older adults as partners in research. Several areas were highlighted for future attention, for example redressing power imbalances. Similarly, a more recent review found that older adults were often positioned as participants versus co-researchers, did not have equal status in the research partnership, were rarely involved in the development of the research question and restricted in the enactment of change. Sustainable collaborations were also rare and marginalised groups such as the disabled or chronically ill were often excluded from participation ( Corrado *et al.,* 2020). 

Reviews of the use of PAR with younger populations have also determined that the principles of PAR were not always realized. One systematic review, exploring youth participatory action research (YPAR) found that only fifteen percent of the 399 papers retrieved involved co-partnering and, of these, almost half excluded co-researchers at the analysis and dissemination stages of the research ( Jacquez *et al.,* 2013). Inconsistency of participation has also been evident in studies involving disabled co-researchers. A review which examined partnered research with disabled persons, aged 5–25 years, identified that few studies reported the exact nature of co-involvement, concluding that there remained scope for methodological research to inform appropriate approaches to public and patient involvement in childhood disability research ( Bailey *et al.,* 2015). 

Although the literature on PAR has been reviewed and critically interpreted, in relation to some groups, this has not occurred with disabled adolescents, indicating a need for this current review. Whilst informative, the review by Bailey & colleagues (2015) was predominantly descriptive in outlining how disabled young persons contributed to research partnerships. We aim to synthesise the available data to interrogate the practices of academic researchers employing a PAR approach when working along-side disabled adolescents.

The objectives of the study are:

•   To scope and critically interpret the use of PAR with disabled adolescents to date. This will include a detailed description of how PAR has been operationalised when working with disabled adolescents and an interrogation of these practices in relation to established PAR principles.

•   To develop a ‘best-practice’ framework to guide researchers when partnering with disabled adolescents in future research.

## Methods

A CIS will be conducted to identify and synthesise the relevant literature to address the review aim. This methodology allows for a diverse range of evidence, including both empirical and non-empirical sources, to be drawn from to develop new understandings in a reflexive and dynamic manner as the review is undertaken ( Ako-Arrey *et al.,* 2016). This methodology was developed by Dixon-Woods *et al.* (2006) to offer a flexible framework in the generation of new theory. We will follow the five steps described by Dixon-Woods *et al.* (2006) in conducting the CIS:

1. Formulation of the review question
2. Searching of the literature
3. Determination of quality
4. Data extraction
5. Interpretive synthesis

### Phase 1: Formulation of the review question

When conducting a CIS, the research question may not be fully defined until the review progresses, although the field of research is declared from the outset ( Yazdani *et al.*, 2015). We broadly define the area of interest as ‘examination of the practices of academic researchers employing a PAR approach when working along-side disabled adolescents’; the preliminary question will be refined at the analysis stage of the review ( Dixon-Woods *et al.,* 2006). As the exact research question will unfold over time, the authors will explicitly document the reasoning associated with these decision-making processes ( Wilson *et al.,* 2014).

### Phase 2: Search strategy

We will conduct both a systematic and manual search of the literature in this study. A systematic search will be undertaken initially. Nine electronic databases will be searched, namely Academic Search Complete, AMED, CINAHL Complete, Education Source, EMBASE, ERIC, MEDLINE, PsychArticles and PsycINFO from the year 1990, using the search terms and strategy detailed in Table 1 and Table 2. In designing the search strategy, a list of Boolean-linked terms was constructed, covering content domains relating to (1) adolescence, (2) disability and (3) participatory action research.

**Table 1.** Search strategy.

Adolescence	&	Disability	&	Participatory action research
• Child* • Adolescen* • Teen* • Youth • Young people • Young person		• Disab* • Impair* • Special needs • Special educational need • Neurodevelopmental disorder • Neurodisabil* • Child Development Disorder • Developmental Delay or Disab* or Disorder • Chronic Condition • Cerebral Palsy • Mental/Intellectual Impair* or Disab* • Down’s Syndrome or Down Syndrome • Fragile X Syndrome or FRAXA or FRAXE or Martin Bell Syndrome • Rett Syndrome • Angelman Syndrome • Prader Willi Syndrome • Williams Syndrome • Fetal/Foetal Alcohol Spectrum Disorder • Communication Disorder • Language Disorder • Speech Sound Disorder • Phonological Disorder • Childhood-Onset Fluency Disorder • Autism Spectrum Disorder or Autism or ASD or Autistic Disorder • Asperger Syndrome • Attention Deficit Disorder or ADD • Attention Deficit Hyperactivity Disorder or ADHD or Hyperactivity Disorder • Learning/Cognitive Impair* or Disab* or Defici* or Difficult* or Disorder* • Motor or Movement Disorder • Developmental Co-ordination Disorder or DCD or Dyspraxia • Tic Disorder • Tourette* • Deaf or Deafness or Hearing Loss or Impair* • Blind or Blindness or Low Vision or Visually Impair* or Visual Impair*		• Action research • Participatory action research • Community-based participatory action research • Participatory research • Public and patient involvement or PPI • Co-researcher • Partnered research • Research partnerships • Research partners • Co-developed research • Co-designed research • Co-constructed research • Collaborative research • Participatory research techniques • Participatory research approach • Inclusive research

**Table 2.** Sample search string.

Academic Search Complete	
S1	TI (Child* OR Adolescen* OR Teen* OR Youth OR “Young person” OR “Young people”) OR AB (Child* OR Adolescen* OR Teen* OR Youth OR “Young person” OR “Young people”)
S2	TI (“Action research” OR “Participatory action research” OR “Community-based participatory action research” OR “Participatory research” OR “Public and patient involvement” OR PPI OR Co-researcher OR “Partnered research” OR “Research partnerships” OR “Research partners” OR “Co-developed research” OR “Co-designed research” OR “Co-constructed research” OR “Collaborative research” OR “Participatory Research Techniques” OR “Participatory Research Approach” OR “Inclusive Research” ) OR AB ( “Action research” OR “Participatory action research” OR “Community-based participatory action research” OR “Participatory research” OR “Public and patient involvement” OR PPI OR Co-researcher OR “Partnered research” OR “Research partnerships” OR “Research partners” OR “Co-developed research” OR “Co-designed research” OR “Co-constructed research” OR “Collaborative research” OR “Participatory Research Techniques” OR “Participatory Research Approach” OR “Inclusive Research” )
S3	TI (Disab* OR Impair* OR “Special needs” OR “Special educational need” OR Neurodevelopmental OR Neurodisabil* OR “Child development disorder” OR "Developmental Delay" OR "Developmental disab*" OR "Developmental Disorder" OR “Chronic Condition” OR “Cerebral Palsy” OR "Mental Impair*" OR "Mental Disab*" OR "Intellectual Impair*" OR "Intellectual Disab*" OR “Down's syndrome” OR "Down Syndrome" OR "Fragile X Syndrome" OR FRAXA OR FRAXE OR "Martin Bell Syndrome" OR "Rett Syndrome" OR "Angelman Syndrome" OR "Prader Willi Syndrome" OR "Williams Syndrome" OR "Fetal Alcohol Spectrum Disorder" OR "Foetal Alcohol Spectrum Disorder" OR "Communication Disorder" OR "Language Disorder" OR "Speech Sound Disorder" OR "Phonological Disorder" OR "Childhood-Onset Fluency Disorder" OR “Autism Spectrum Disorder” OR Autism OR ASD OR “Autistic Disorder” OR “Asperger Syndrome” OR “Attention Deficit Disorder” OR ADD OR “Attention Deficit Hyperactivity Disorder” OR ADHD OR “Hyperactivity Disorder” OR “Learning Impair*” OR “Learning Disab*” OR “Learning Defici*” OR “Learning Difficult*” OR “Learning Disorder*” OR “Cognitive Impair*” OR “Cognitive Disab*” OR “Cognitive Defici*” OR “Cognitive Difficult*”OR “Cognitive Disorder*” OR “Motor Disorder” OR “Movement Disorder” OR “Developmental Co-ordination Disorder” OR DCD OR Dyspraxia OR “Tic Disorder” OR Tourette* OR Deaf OR Deafness OR “Hearing Loss” OR “Hearing Impair*” OR Blind or Blindness OR “Low Vision” OR “Visually Impair*” OR “Visual Impair*” ) OR AB ( Disab* OR Impair* OR “Special needs” OR “Special educational need” OR Neurodevelopmental OR Neurodisabil* OR “Child development disorder” OR "Developmental Delay" OR "Developmental disab*" OR "Developmental Disorder" OR “Chronic Condition” OR “Cerebral Palsy” OR "Mental Impair*" OR "Mental Disab*" OR "Intellectual Impair*" OR "Intellectual Disab*" OR “Down's syndrome” OR "Down Syndrome" OR "Fragile X Syndrome" OR FRAXA OR FRAXE OR "Martin Bell Syndrome" OR "Rett Syndrome" OR "Angelman Syndrome" OR "Prader Willi Syndrome" OR "Williams Syndrome" OR "Fetal Alcohol Spectrum Disorder" OR "Foetal Alcohol Spectrum Disorder" OR "Communication Disorder" OR "Language Disorder" OR "Speech Sound Disorder" OR "Phonological Disorder" OR "Childhood-Onset Fluency Disorder" OR “Autism Spectrum Disorder” OR Autism OR ASD OR “Autistic Disorder” OR “Asperger Syndrome” OR “Attention Deficit Disorder” OR ADD OR “Attention Deficit Hyperactivity Disorder” OR ADHD OR “Hyperactivity Disorder” OR “Learning Impair*” OR “Learning Disab*” OR “Learning Defici*” OR “Learning Difficult*” OR “Learning Disorder*” OR “Cognitive Impair*” OR “Cognitive Disab*” OR “Cognitive Defici*” OR “Cognitive Difficult*”OR “Cognitive Disorder*” OR “Motor Disorder” OR “Movement Disorder” OR “Developmental Co-ordination Disorder” OR DCD OR Dyspraxia OR “Tic Disorder” OR Tourette* OR Deaf OR Deafness OR “Hearing Loss” OR “Hearing Impair*” OR Blind or Blindness OR “Low Vision” OR “Visually Impair*” OR “Visual Impair*”)
S4	S1 AND S2 AND S3

It was decided to narrow the population under research to adolescents with a diagnosis of neurodevelopmental disability, excluding those with mental health difficulties and those living with chronic medical conditions; whilst it is acknowledged that similar barriers to participation may be experienced by these adolescents, such as stigma, there are specific issues associated with a diagnosis of neurodevelopmental disorder. Therefore, search terms have been developed based on the DSM-5 classification of neurodevelopmental disorder and related reviews ( Iemmi *et al.,* 2016; Levy *et al.,* 2020; Reichow *et al.,* 2019). The authors are cognisant that the search strategy includes impairment-focused terms in conflict with our chosen social relational perspective of disability. This pragmatic decision was made given the broad range of disciplines publishing in our review area, including many health disciplines where the use of these terms is common practice. The screening process will occur over several stages with strict adherence to the inclusion-exclusion criteria to maximise the relevancy of the retrieved data, in line with the preliminary research question.

The inclusion criteria for the systematic review are:

- Papers, theses and reports published in the English language only.
- Published from 1990 to present day.
- Qualitative, quantitative and mixed-methods studies will be included.
- Studies involving disabled adolescents in the role of co-researcher, within a PAR approach.
- Adolescence is defined as the period between 13–18 years of age, as operationalised previously ( Kelleher *et al.,* 2012). Studies which traverse different age-groups will be included/excluded according to the mean age of the co-researchers.
- The context will be further refined to co-researchers with neurodevelopmental disorders, to include those with intellectual disability, developmental delay, cerebral palsy, visual or hearing impairment, motor disorders, autism spectrum disorder, specific language disorders, specific learning disability and/or attention deficit (hyperactivity) disorder. Please refer to Table 1 for further details.

The exclusion criteria include:

- Studies published prior to 1990.
- Papers, theses or reports not published in the English language.
- Studies which involved adult co-researchers or those under the age of 13 years. Where the age-range of co-researchers cuts across younger or older age-groups, papers will be excluded if the mean age of co-researchers is outside of the defined 13–18 years age-range.
- Studies which involved adolescents as participants versus co-researchers
- Studies which involved adolescents with mental health difficulties or chronic medical conditions.
- Grey literature, excluding theses and reports, will be excluded from this initial search but may be purposively searched, at the later analysis stage of the study, to challenge/support emerging concepts, as described by Dixon-Woods & colleagues (2006).

Electronic search results will be imported into Rayyan QCRI software, duplicates will be removed, and title and abstract screening will be conducted by two authors independently (FM, JP, KR, AG), with FM screening all papers and JP, KR and AG each screening one-third of papers. The same process will be used at the next stage of full paper reading and screening. Uncertainties will be discussed by the authors until a consensus is reached. The reference lists of the included papers will be scanned to identify other potentially relevant material. Reflexivity will occur throughout this phase of the review, culminating in the selection of the most relevant articles to inform the aims of the review ( Wilson *et al.,* 2014). A teamwork approach will be used, drawing on multiple perspectives to support decision-making and enhance rigour ( Barry *et al.,* 1999). 

### Phase 3: Determination of quality

Dixon-Woods *et al.* (2006) discussed the difficulty in screening papers by quality due to the multiple forms of data elicited. They determined, instead, to focus on the relevancy of papers, excluding only those papers that were “ *fatally flawed*” (p.4). They reported five questions to employ in the scrutinization of the quality of papers, as follows:

1. Are the aims and objectives of the research clearly stated?
2. Is the research design clearly specified and appropriate for the aims and objectives of the research?
3. Do the researchers provide a clear account of the process by which their findings could be reproduced?
4. Do the researchers display enough data to support their interpretations and conclusions?
5. Is the method of analysis appropriate and adequately explicated?

Two researchers will independently appraise all included papers / data sources against the five questions above (FM, JP, KR, AG). FM will appraise all papers and the other authors will appraise one third of included papers each. We will exclude only those papers which fail to comply with the methodological expectations outlined. Disagreements will be resolved through team discussion. The decision to exclude papers on the basis of ‘fatal flaws’ will occur within the synthesis phase of the review.

### Phase 4: Data extraction

A custom data extraction template will be developed to describe the characteristics of the papers for analysis (authors, publication date, origin, type of study, methodology), the demographics of co-researchers, the level of co-researcher involvement at the different stages of the PAR process, the experience/reflections of co-researchers (agency, equality, respect, personal benefits, challenges), possible social barriers to full participation for team members (environmental, cultural and attitudinal), adaptations made to facilitate participation, the outcomes of collaborative research practices (main findings and action outputs), and the experience/ reflections of the academic researcher.

### Phase 5: Interpretative synthesis

At this phase of the review Dixon-Woods *et al.* (2006) suggest that the data is treated in the same way as a qualitative synthesis; the CIS approach is grounded in meta-ethnography, a common approach to synthesis of qualitative studies. Building on the work of Noblit & Hare (1988), three key phases are described in the analysis and synthesis of the data ( Toye *et al.,* 2014), including: 1) exploration of how studies are related, 2) translation of the studies and 3) synthesis of translations (p.3).

In exploring how studies are related, repeated reading of the papers will occur and the authors will independently identify concepts within and between papers ( Cahill *et al.,* 2018) relating to the research aim. NVivo 12 Pro software will be used to document concepts from individual studies and to map the evolution of concepts. Through discussion, these early concepts will be further explored and refined, from our differing perspectives. In the next stage, the concepts will be grouped into conceptual categories by the individual reviewers and then further interpreted through collaborative enquiry ( Toye *et al.,* 2014), with agreement reached on categorisation. The team approach to analysis and synthesis will also allow the first author, an early-stage researcher, to draw on the expertise of more experienced team members as recommended in qualitative synthesis approaches ( Cahill *et al.,* 2018). Additionally, for the purposes of this review, and future doctoral research by the lead author (FMcD), an adolescent co-researcher team will be established; the co-researcher team will be involved at the analysis and synthesis stages of this review.

Dixon-Woods *et al.* (2006) define the interpretation of evidence from the original studies into new concepts as the generation of ‘ *synthetic constructs*’ (p. 6), akin to third order constructs in meta-ethnography. As data is analysed and hypotheses begin to emerge, purposive searching will be conducted by the lead author to identify additional sources to support analysis. Potential avenues of purposive searching may include linking with experts or a search of web-sites to source data not elicited from the initial systematic search ( Depraetere *et al.,* 2020). The adolescent co-researcher team will be involved in the formulation of the emerging concepts, as experts in the lived experience of disability, with the purpose of defining meaningful constructs. Further synthesis of the data will allow for the generation of theory related to the phenomenon of interest, participatory action research. To support this process, the CIS approach demands that the evidence is critiqued, recognising anomalies within the data, considering the potential underlying influencers of assumptions made and identifying possible deficiencies in the resultant theories proffered ( Dixon-Woods *et al.,* 2006). 

To ensure the trustworthiness of the theory generated through this CIS, the lead author will maintain a reflective diary to map her interaction with the data, being transparent in her reasoning at all stages of the process of analysis, discussing and debating emerging ideas and assumptions with the co-authors. Also, as an academic researcher, who will be co-researching with disabled adolescents, the lead author will be actively reflexive in examining her own beliefs and attitudes in line with the emerging constructs, to identify and manage potential bias which could otherwise impact the prospective study. The recurring themes that emerge will be interrogated against the principles of PAR to evaluate current practices building on these to generate a ‘best practice’ framework to inform future practice when partnering with disabled adolescents as co-researchers. 

## Conclusion

In conclusion, the authors will complete a CIS to interrogate the practices of academic researchers employing a PAR approach when working along-side disabled adolescents. In 1989, the UN Convention of Rights of the Child called for the voice of children and adolescents to be heard. Co-engagement in research is one avenue to facilitate this. However, although PAR has been used extensively in the intervening 30 years, there has been little analysis of the extent to which PAR processes are applied in practice when researching alongside disabled adolescents. Critical engagement with the existing literature will occur through this CIS, allowing for new understandings on the use of PAR with this group to be formulated. A best-practice framework, to support academic researchers in working with this marginalised group, will be a key output of the review with the aim of optimising the forum for disabled adolescents to participate as co-researchers.

## Data availability

No data are associated with this article.
