# Supplementary material for: Exploring the co-involvement of disabled adolescents in participatory action research; protocol for a critical interpretative synthesis
Source: HRB Open Res. 2021 Jul 22;4:79. [Version 1] doi: 10.12688/hrbopenres.13343.1 (PMC8689409; doi:10.12688/hrbopenres.13343.1)
Supplement: Peer review history

# Peer review: Reviewer response for version 1

**DOI:** 10.21956/hrbopenres.14531.r30978

**Published:** 2021-12-20

**Authors:** Martha L. P. MacLeod

**Affiliations:** 1 School of Nursing, University of Northern British Columbia, Prince George, BC, Canada

This is a protocol for a critical interpretive synthesis (CIS) review of participatory action research (PAR) with disabled adolescents. It is positive to see that the CIS approach to the review is consistent with the subject of the review (PAR). The method is described well, and appropriately.

 The questions the authors may wish to consider relate to the conceptualization of the adolescent researchers, embracing the power realities within research teams, and the criteria for determining quality of the retrieved documents/data sources:

- The gaze in the review is on the practices of academic researchers in conducting PAR. It might be helpful to also turn the gaze on these academic researchers themselves, to assess what they learned about themselves when working with adolescent researchers (and what they discovered). For instance, did the academic researchers gain skills in self-reflection or new ways of understanding the adolescent researchers?
- Determination of quality through the 5 questions is to be done on the processes of the research. In a CIS review, the epistemological coherence of this form of quality assessment merits reconsideration. That is, the positivist bent of the quality assessment questions towards the notion that if the process is right, and the description is right, the findings must be credible, should be questioned. Question 4, about displaying enough data, may be moot if there is little coherence with the interpretation or that the themes reflect trivial observations. While the process-focus for determining quality is conventional in knowledge synthesis approaches, it may be worthwhile to take another look while undertaking this particular CIS.
- The adolescent researchers are going to be involved in the analysis and synthesis stages. It may be worthwhile involving them at earlier stages as well, and in decision-making throughout if they are really to be co-researchers.

Is the study design appropriate for the research question?

Yes

Is the rationale for, and objectives of, the study clearly described?

Yes

Are sufficient details of the methods provided to allow replication by others?

Yes

Are the datasets clearly presented in a useable and accessible format?

Not applicable

Reviewer Expertise:

participatory action research; knowledge mobilization

I confirm that I have read this submission and believe that I have an appropriate level of expertise to confirm that it is of an acceptable scientific standard.

# Peer review: Reviewer response for version 1

**DOI:** 10.21956/hrbopenres.14531.r30983

**Published:** 2021-12-08

**Authors:** Mary Casey

**Affiliations:** 1 UCD School of Nursing, Midwifery and Health Systems, Health Sciences Centre, University College Dublin, Dublin, Ireland

The purpose of the article is to set out a protocol to undertake a critical interpretative synthesis on the use of Participatory Action Research (PAR) with disabled adolescents to date. This will include a detailed description of how PAR has been operationalised when working with disabled adolescents and an interrogation of these practices in relation to established PAR principles.

 There is extant literature on the quality of action research and identification of the key principles of action research – of which PAR is one typology. Coghlan and Shani suggest that demonstrating the quality of action research studies can be achieved by: i) providing knowledge of the practical and academic context of the project; ii) enabling participants to become co-researchers; iii) undertaking cycles of action and reflection as the project is being implemented and knowledge is being co-generated; and iv) creating outcomes that are both achievable and sufficiently strong for theory development about the topic itself, or the process of undertaking the change or the actual change. ^1^ It is difficult to see how this review on the ‘use of PAR’ can be undertaken without some reference to these principles or quality indicators.

 A second stated objective is ‘to develop a ‘best-practice’ framework to guide researchers when partnering with disabled adolescents in future research’. Building on existing criteria for best practice when using an action research approach such as PAR seems to be a good place to start.

 Other than the above point, this is a very well-written article.

Is the study design appropriate for the research question?

Yes

Is the rationale for, and objectives of, the study clearly described?

Yes

Are sufficient details of the methods provided to allow replication by others?

Yes

Are the datasets clearly presented in a useable and accessible format?

Yes

Reviewer Expertise:

Action Research, Organisation Development, Health Systems Research

I confirm that I have read this submission and believe that I have an appropriate level of expertise to confirm that it is of an acceptable scientific standard, however I have significant reservations, as outlined above.

## References

[1] : Conducting Action Research for Business and Management Students.2018; 10.4135/9781529716566 10.4135/9781529716566

# Peer review: Reviewer response for version 1

**DOI:** 10.21956/hrbopenres.14531.r29915

**Published:** 2021-08-23

**Authors:** Karen R. Fisher

**Affiliations:** 1 Social Policy Research Centre, UNSW Sydney, Sydney, NSW, Australia

The article is the protocol for a review of participatory action research with disabled adolescents. It is well described and adds to the evidence base. Suggestions for improvement are below:

- The first paragraph should refer to the UNCRPD in addition to  UNCRC, given its relevance to disabled adolescents and that it is a newer Convention.
- The background introduction could refer more to disability inclusive research, which is a substantial literature overlapping with PAR, including with adolescents.
- The justification of 13-18 should be further explained or explored since life transitions of disabled adolescents is often interpreted as older than 18, especially young people with learning disabilities.
- Acknowledgment that the terminology is location specific would be helpful for an international audience. For example, person first language is not just contested in relation to the type of disability model but is also the international policy position of people with disabilities in various countries, including agreements reached during the CRPD process.
- The language of 'co-researcher' could be explained, or at least acknowledged that it is also contested (co- being only applied to the non-academic researcher).
- The role of co-researchers in the review should be explained – active researchers in which steps, advisors, or not at all (which, given the topic, it would be difficult to justify).
- Review to 1990 seems a very long period. It should be justified and explained why it is necessary and how it is feasible.

Is the study design appropriate for the research question?

Yes

Is the rationale for, and objectives of, the study clearly described?

Yes

Are sufficient details of the methods provided to allow replication by others?

Yes

Are the datasets clearly presented in a useable and accessible format?

Not applicable

Reviewer Expertise:

disability policy research

I confirm that I have read this submission and believe that I have an appropriate level of expertise to confirm that it is of an acceptable scientific standard, however I have significant reservations, as outlined above.
